# Change of Title: *Microarrays* Becomes *High-Throughput*

**DOI:** 10.3390/ht6030010

**Published:** 2017-07-19

**Authors:** Massimo Negrini

**Affiliations:** Department of Morphology, Surgery and Experimental Medicine, University of Ferrara, 44121 Ferrara, Italy; ngm@unife.it

MDPI’s journal *Microarrays* released its first volume in 2012. Since then, the journal has published 129 articles on the topic of microarrays, including their applications, analysis and new developments.

The array technology saw its infancy in the mid–late 1970s, when the work of Grunstein and Hogness [1] and Gergen and colleagues [2] allowed the generation of a high-dimensional collection of bacterial DNA on filter-based arrays. However, the origin of what we consider modern microarrays can be placed in the mid 1990s, when the work by Schena, Davis and Brown at the Stanford University was published in top scientific journals [3,4]. Since then, the development of oligonucleotide synthesis, fluorescence detection methods and advances in automation processes enabled the use of microarrays in a large number of biological and medical studies aimed at investigating gene expression, DNA methylation, DNA copy number variations, biomarker discovery, and pathogen detection, quickly becoming the main high-throughput method in many laboratories across the globe [5,6,7,8]. Protein arrays, tissue arrays as well as many other specialized arrays have also been developed and widened the potential of this approach [9,10].

The advent of next-generation sequencing (NGS) added a new powerful tool to the technological resources available for genome-wide investigations, thus widening the potential of high-throughput investigations in biology and medicine [11,12,13,14].

To keep pace with these constant technological changes, the journal has chosen to change its name: *Microarrays* will be renamed *High-Throughput*.

Not only the name of the journal is being updated but also its scope, covering any high-dimensional approaches and the accompanying computational developments that enable the analysis and interpretation of large volumes of data generated in the Omics era.

On behalf of the Editorial Board and the Editorial Office, I wish to thank all authors, reviewers and external editors for their support to *Microarrays* during these years, and welcome them to join us in developing *High-Throughput* to its full potential.
